# Ecology Drives the Worldwide Distribution of Human Diseases

**DOI:** 10.1371/journal.pbio.0020141

**Published:** 2004-06-15

**Authors:** Vanina Guernier, Michael E Hochberg, Jean-François Guégan

**Affiliations:** **1**Génétique et Évolution des Maladies InfectieusesMontpellierFrance; **2**Unité Expertise et Spatialisation des Connaissances en EnvironnementMontpellierFrance; **3**Équipe Génétique et Environnement, Institut des Sciences de l'Évolution de MontpellierUniversité Montpellier II, MontpellierFrance; **4**National Center for Ecological Analysis and Synthesis, University of CaliforniaSanta Barbara, CaliforniaUnited States of America

## Abstract

Identifying the factors underlying the origin and maintenance of the latitudinal diversity gradient is a central problem in ecology, but no consensus has emerged on which processes might generate this broad pattern. Interestingly, the vast majority of studies exploring the gradient have focused on free-living organisms, ignoring parasitic and infectious disease (PID) species. Here, we address the influence of environmental factors on the biological diversity of human pathogens and their global spatial organization. Using generalized linear multivariate models and Monte Carlo simulations, we conducted a series of comparative analyses to test the hypothesis that human PIDs exhibit the same global patterns of distribution as other taxonomic groups. We found a significant negative relationship between latitude and PID species richness, and a nested spatial organization, i.e., the accumulation of PID species with latitude, over large spatial scales. Additionally, our results show that climatic factors are of primary importance in explaining the link between latitude and the spatial pattern of human pathogens. Based on our findings, we propose that the global latitudinal species diversity gradient might be generated in large part by biotic interactions, providing strong support for the idea that current estimates of species diversity are substantially underestimated. When parasites and pathogens are included, estimates of total species diversity may increase by more than an order of magnitude.

## Abstract 2

Comparative analyses reveal that human pathogens increase towards the equator and that the relationship is linked to climate - this has important implications for global biodiversity, public health and environmental epidemiology

## Introduction

Generally, the number of plant and animal species declines as one moves away from the equator (Pianka 1966; Stevens 1989, 1992; Rohde 1992; Brown 1995; Kaufman 1995; Rosenzweig 1995; Roy et al. 1998; Huston 1999; Chown and Gaston 2000; Hawkins and Porter 2001). This pattern, known as the latitudinal species diversity gradient, has been documented for many contemporary taxonomic groups (see Brown 1995; Rosenzweig 1995; Gaston and Blackburn 2000; Allen et al. 2002; Stevens et al. 2003). Over 30 hypotheses have been proposed to explain it (Rohde 1992), and it is only over the past several years that the most credible candidates have been identified; these are hypotheses related to area, energy, and time (Gaston and Blackburn 2000; Rahbek and Graves 2001) and to habitat heterogeneity and geometric constraints (Rahbek and Graves 2001).

The vast majority of studies exploring the latitudinal species diversity gradient have focused on free-living organisms, such as herbivores, mammals, and angiosperms, and with rare exception (Hillebrand et al. 2001; Curtis et al. 2002; Nee 2003), none has examined large-scale latitudinal species diversity patterns of pathogenic microorganisms. Biotic interactions such as parasitism, predation, and symbiosis have been often invoked as a causal mechanism for the gradient (see Rohde 1992), but no serious attempts have been made to quantify its importance to biodiversity. Parasitic and infectious diseases (PIDs), in particular, could prove to be key in understanding large-scale patterns of species diversity on Earth since they comprise a major part of total biological diversity (Combes 1995; Poulin 1998). Moreover, our understanding of human diseases and the existence of complete data sets provide an incomparable opportunity to explore the existence of a relationship between PID species richness and latitude, and to identify the determining factors of this latitudinal gradient.

**Figure 1:**
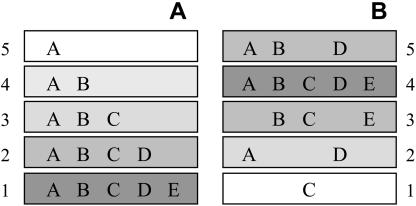
The Spatial Organization of Species Letters represent different PID species. Numbered rectangles represent different countries or areas. (A) Nested organization of species. Applying Diamond's theory, we here distinguish (1) “high-S” species, like species E, which are exclusively confined to the most species-rich communities; and (2) “tramps,” like species A, which occur mostly in richer communities but also in species-poor communities (e.g., measles, which is found in virtually every country). Thus, this nested pattern implies that some pathogens are restricted to the tropics, while others, more ubiquitous species, are widely and regularly distributed all over the world. (B) Random distribution of species, where no spatial organization occurs (see also Materials and Methods).

In recent years, research into nonrandom organization in parasite communities has turned, e.g., to the possible existence of nestedness. Nested structure is a hierarchical organization of species composition in which assemblages with successively lower species richness tend to be nonrandom subsets of richer assemblages (Hanski 1982; Patterson and Atmar 1986; Patterson and Brown 1991; Poulin and Guégan 2000). Some species are widely distributed and occur in many communities, whereas other species have more restricted distributions and occur only in a subset of the richest samples (Figure 1). When analysing the most important mechanisms responsible for generating nestedness, Wright et al. (1998) cited four candidate factors: random sampling, area, isolation, and habitat type. In the present study we seek an answer to the following question: To what extent is the global distribution of human pathogens specified by the properties of the physical environment or the organism itself, and to what extent does it depend on chance events?

We examine the global spatial distribution of species richness for human PIDs, and test the hypothesis that human diseases follow a latitudinal species richness gradient, with low latitudes being the richest zones in pathogen species diversity. We then test two additional propositions: (i) PID assemblages show nested species patterns along latitudinal gradients, i.e., PIDs present at northern latitudes are also present in larger PID assemblages of equatorial zones, and (ii) PID assemblages may be strongly influenced by environmental climatic forces.

## Results

### The Latitudinal Gradient of Species Richness for Pathogens

**Figure 2:**
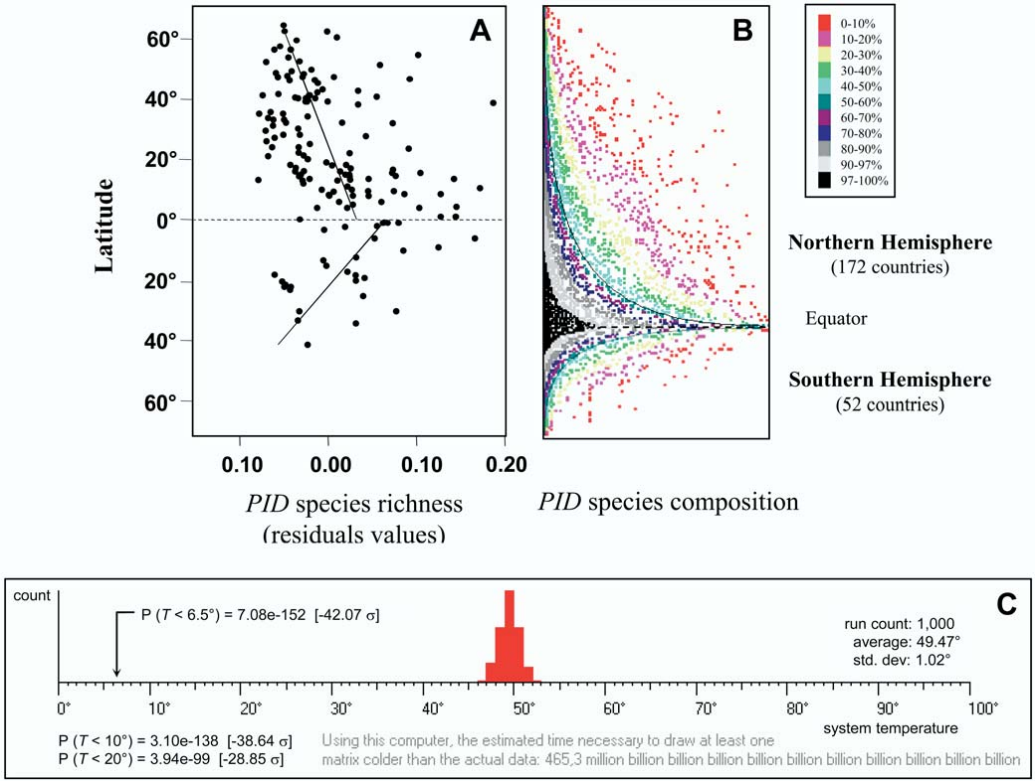
The Latitudinal Gradients of PID Species (A) Relationship between PID species richness and latitude across the two hemispheres. Linear relationships between PID species richness and latitude (dotted lines) are highly significant (*F* = 12.29, *df* = 29, *p* = 0.0015 and *F* = 18.01, *df* = 130, *p <* 0.0001 for Southern and Northern hemispheres, respectively). No difference in disease species richness with latitude across the two hemispheres was observed (interaction: *F* = 2.68, *df = 159*, *p* = 0.1036). Residuals of PID species richness on the *y* axis were extracted from minimal models controlling for the effects of confounding factors on PID species diversity estimates (see Materials and Methods). Locally weighted regression (tension 0.5) did not change the general linear shape. Latitude is expressed in minute degrees. (B) Presence/absence matrix for the 229 distinct PID species across the hemispheres. The figure was generated by the Nestedness Temperature Calculator (see Atmar and Patterson 1995). The distribution is nonsymetrical because of the 224 studied countries, 172 countries are found in the Northern hemisphere versus only 52 in the Southern one. (B) indicates that PID species diversity decreases as one moves northwards or southwards from the equator. The black exponential curves are the occurrence boundary lines (see Materials and Methods). The color scale indicates the nonuniform probability of state occupancy among all of the cells of the matrix, i.e., the probability of encountering a species as function of its position in the matrix. Black cells are highly predictable presences, whereas red cells are unexpected presences. (C) Monte Carlo–derived histogram after 1,000 permutations. The histogram represents the 1,000 values obtained after Monte Carlo permutations. The average theoretical value under the null hypothesis is compared to our real value, to assess the likelihood that the parent matrix was nonrandomly generated. The probability is highly significant (*p* < 0.0001), confirming that the spatial organization of PID species richness on the largest scale matches the nested species subset hierarchy illustrated in Figure 1A. The symmetrical Gaussian distribution indicates that 1,000 permutations are enough to obtain reliable variance estimates for probability calculations.

After correcting for cofactors (i.e., area and socio-demographic, physical, and environmental parameters) that could influence the relationships between latitude and PID species richness, we still found that species richness in human pathogens is strongly correlated with latitude (Table 1). On average (seven times out of ten), tropical areas harbor higher pathogen species diversities compared to more temperate areas. Figure 2A illustrates the change in PID species diversity with latitude across the two hemispheres.

**Table 1:**
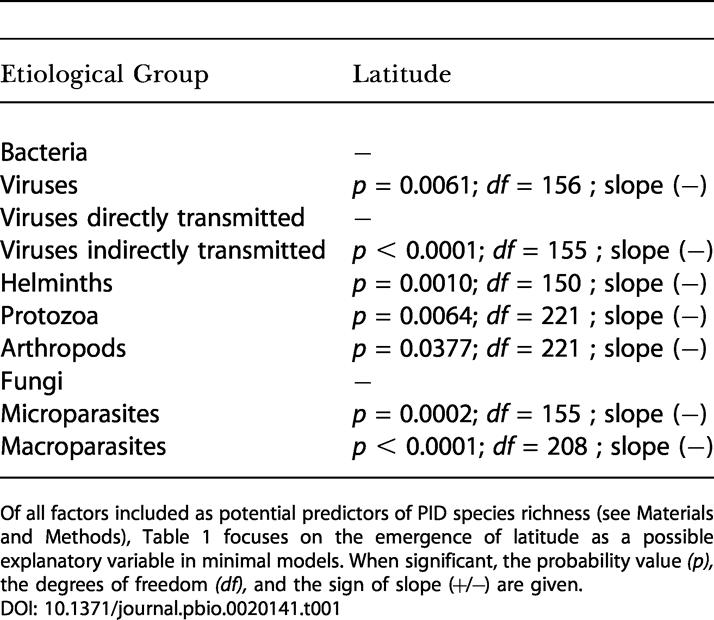
Minimal Models for Latitude Explaining PID Species Richness of Etiological Groups

Of all factors included as potential predictors of PID species richness (see Materials and Methods), Table 1 focuses on the emergence of latitude as a possible explanatory variable in minimal models. When significant, the probability value *(p),* the degrees of freedom *(df),* and the sign of slope (+/−) are given

### The Nested Organization of Pathogen Species over Large Scales

Monte Carlo analyses confirmed an overall nested species pattern of global distribution in PID species richness (*N_s_* = 2,481.4, R_0_ and R_1_ procedures, *p* < 0.0001) and showed diversity to be strongly nested, with some anecdotal differences across the different groups of etiological agents (all groups, *p* < 0.0001, except for vector-borne viruses, with the R_1_ procedure [*N_s_* = 1,787, *p* = 0.0015]). When considering the Northern and Southern hemispheres separately, both were highly nested (R_0_ and R_1_ procedures, *N_s_* = 6,602, *p* < 0.0001 and *N_s_* = 1,230, *p <* 0.0001, respectively). This was confirmed by the R_00_ procedure used by the Nestedness Temperature Calculator program (Atmar and Patterson 1995), which provides a useful graphic representation of the results (Figure 2B), showing that PID species diversity decreases as one moves northwards or southwards from the equator (*F* = 28.2307, *df* = 161, *p* < 0.0001). The occurrence boundary lines (black exponential curves) were fitted by nonlinear regression (*y* = 1.51 + 20.01e^-0.29x^ and *y* = 1.65 + 35.87e^-0.36x^ for Northern and Southern hemispheres, respectively). Results from Monte Carlo simulations confirmed that our nested matrix was nonrandomly generated (*p* < 0.0001) (Figure 2C). The spatial organization of PID species richness on the largest scale matches the nested species subset hierarchy illustrated in Figure 1A.

Thus, pathogen species that compose a depauperate community in temperate conditions statistically constitute a proper subset of those occurring in warmer conditions, and evidence of pathogen species occurring in temperate areas but not in tropical ones was rare or anecdotal. It should be noted that, at this large spatial scale, our study demonstrates a nested pattern in PIDs*,* with a progression of species richness from polar regions to the equator, indicating that nestedness is strongly associated with latitude (see Figure 2B). But this does not contradict the fact that some pathogens may be strict endemics of more temperate areas (e.g., Lyme disease).

### The Effect of Climatic Variables on Biodiversity

Latitude is a proxy variable for a wide range of covarying bio-climatic factors and in itself has no meaning regarding factors potentially affecting species diversity. We therefore investigated the relationship between pathogen diversity and individual climatic variables reflected in the composite variable “latitude” (Table 2). Results show significant positive correlations between pathogen species richness and the maximum range of precipitation after Bonferroni multiple corrections for all six of the PID taxa considered: bacteria (r = 0.3545, *df =* 213, *p <* 0.0001), viruses directly transmitted from person to person (r = 0.2350, *df* = 215, *p <* 0.0001), viruses indirectly transmitted via a vector (r = 0.3575, *df* = 215, *p <* 0.0001), fungi (r = 0.3554, *df* = 216, *p <* 0.0001), protozoa (r = 0.3744, *df* = 216, *p <* 0.0001), and helminths (r = 0.4270, *df* = 215, *p <* 0.0001). On the other hand, the relationship between PID species richness and monthly temperature range was only significant for three groups of pathogens: bacteria (r = 0.3016, *df* = 213, *p <* 0.0001), directly transmitted viruses (r = 0.2142, *df* = 214, *p* = 0.0015), and helminths (r = 0.2590, *df* = 213, *p* = 0.0001). In contrast to previous results (Allen et al. 2002), we found no significant relationship between PID species richness and mean annual temperature. Finally, only the relationship between bacteria species richness and mean annual precipitation was significant (*r* = 0.1987, *df* = 213, *p* = 0.0034). Very little difference was observed among hemispheres concerning these relationships (data not shown).

**Table 2:**
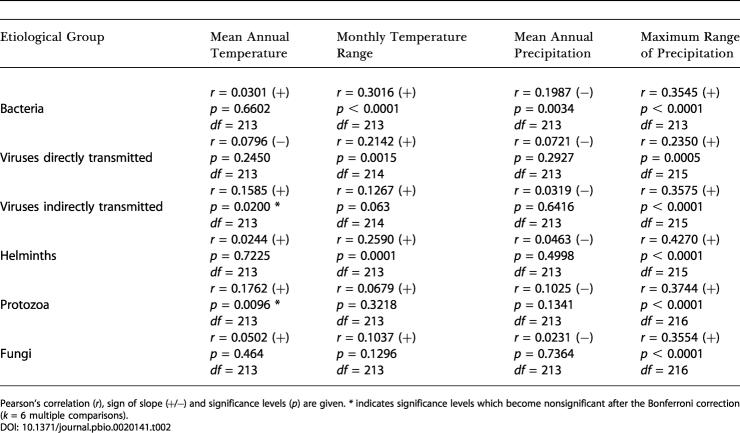
Relationship Between PID Species Richness by Etiological Group and Four Bio-Climatic Factors

Pearson's correlation (*r*), sign of slope (+/−) and significance levels (*p*) are given. * indicates significance levels which become nonsignificant after the Bonferroni correction (*k* = 6 multiple comparisons)

Taken together, these findings indicate that the species richness of human pathogens, their spatial distribution and organization on a large scale, the maximum range of precipitation, and, to a lesser extent, monthly temperature might be intimately connected in generating the observed pattern of disease diversity.

## Discussion

To our knowledge, this is the most comprehensive report of how PID species richness varies with latitude and the ecological factors behind observed trends. Our results support previous studies in showing that species diversity increases as one proceeds from the poles to the equator (Pianka 1966; Stevens 1989; Rohde 1992; Brown 1995; Rosenzweig 1995; Chown and Gaston 2000). This similarity in the patterns of PID species and free-living organisms suggests that common mechanisms are at work. Regardless of whether PID richness simply tracks host diversity or, rather, is determined to a greater extent by exogenous factors, our analyses indicate that the most likely explanation for these patterns is the climatically-based energy hypothesis, i.e., that energy availability generates and maintains species richness gradients (Rohde 1992; Gaston and Blackburn 2000; Allen et al. 2002; Hawkins et al. 2003). Many studies have identified correlations between gradients in species diversity and variation in climate (Hill et al. 1999). Climate, in turn, largely determines the species of plants and animals that live in those areas. According to our results—and in contrast to the results of Allen and colleagues (2002), who showed that environmental temperature was the best predictor of species diversity for terrestrial, freshwater, and marine ectotherm taxa—the maximum range of precipitation is highly correlated with the latitudinal gradient of pathogen species, with diversity significantly increasing with this climate-based factor. Interestingly, the annual variation of precipitation around the mean (and not the mean itself) was the best predictor overall of pathogen species distribution. This suggests that pathogen species, their vectors, or their hosts tend to be adapted to regions having more contrasted wetness and dryness conditions through the year (i.e., in tropical regions). Many parasites obviously require water or humid conditions to complete their life cycle, e.g., vector-borne diseases. So, the physical factor of precipitation variation may affect parasitic and infectious microorganism diversity, if the biological cyclicity of a variety of parasitic and infectious stages have adapted to the variability of precipitation. This might be why “latitude” does not appear in the minimal generalized linear models (GLIMs) for explaining the richnesses of bacteria, directly transmitted viruses, and fungi, these taxa being “internal” to the host, so less directly affected by environmental variability. Moreover, these taxa may more readily spread over longer distances via their hosts, and this should minimize the impact of environmental conditions. In contrast, taxa with “external” stages, like helminths or vector-transmitted pathogens, are more influenced by their environment. Nevertheless, other causes might explain why certain taxa do not conform to the general pattern, notably (1) the absence of possible explanatory variables in the GLIMs, (2) missing or imprecise information due to the large scale of our study, or (3) the real absence of correlations between the spatial distributions of certain taxonomic groups and the variables considered here.

All three nestedness models (see Materials and Methods) explained some of the variation in pathogen species across latitudes. Distance and isolation from pathogen species–rich regions in the tropics may sort PID species by their extinction–colonization dynamics (Lomolino 1996). In addition, the availability of new hosts and reservoirs, passive sampling, and probabilistic filters screening species with particular characteristics (local habitat suitabilities, differential colonization capacities of species, and sustainability of viable populations within their environment) may further limit PID species (Wright et al. 1998) and thus strongly affect the spatial organization of PID species. Nestedness might in fact be an inevitable second-order consequence of the same factors that cause variation in species richness and range size (Gaston and Blackburn 2000).

In addition, our results suggest that total species diversity on the planet might be substantially underestimated, especially because inventories generally focus attention on the most charismatic groups (Shaw and Hochberg 2001), and little is known about the biodiversity of microorganisms associated with each considered group of organisms, i.e., hosts (Ashford and Crewe 1998; Ashford 2000; Nee 2003). Based on a single host species, humans, we estimate that true tropical pathogen species diversity is greater than current estimates by a factor of about 22 in the Northern Hemisphere and about 37 in the Southern Hemisphere. If our work is representative of other (host) species, diversity may be currently underestimated by more than an order of magnitude, and based on our findings, this differential should increase as one goes from temperate to tropical latitudes. Our work quantitatively demonstrates that parasitic and pathogenic organisms, as representatives of biotic interactions, strongly amplify the general latitudinal gradient in species richness. The smallest organisms that have been neglected by science could very well be the biggest in generating the observed diversity pattern.

The demonstration that parasitic and infectious organisms in humans do not constitute random assemblages at large spatial scales, but rather that many types of microorganisms show a predictable geographical distribution over the planet, could have important implications for public health policies. Our results show that climatic factors are of primary importance in explaining the occurrence and diversity of human pathogens, suggesting that global climate change might have cascading effects regarding the risks of PIDs. For instance, if specific temperate areas were to become more tropical, our results suggest that PID species and their associated vectors/reservoirs would be likely to colonize these changed areas. This would imply a progressive dissolution of the latitudinal effect and of the nested hierarchical structure as observed in the present study as pathogen species became more globally distributed. There is some recent evidence for this hypothesis (see Lindgren and Gustafson 2001).

Other variables are indeed important in explaining global-scale patterns of human pathogens (e.g., modernization, urbanization, and pauperization, especially in developing countries). Thus, we do not mean to imply that latitude and surrogate variables are the only ones affecting PID species richness. Nevertheless, our results challenge the conventional wisdom that socio-economic conditions are of preponderant importance in controlling or eradicating diseases. These considerations indicate that a better understanding of PID species diversity and community dynamics in a changing world will be one of the major challenges in environmental epidemiology in the future.

## Materials and Methods

### 

#### Presence/absence matrix

We compiled data on PID occurrence for a total of 332 different human pathogens, including bacteria, viruses, fungi, protozoa, and helminths distributed across 224 nations. Epidemiological data on PID species were extracted from the Global Infectious Diseases and Epidemiology Network database (http://www.cyinfo.com).The presence/absence matrix for the 229 distinct PIDs (after elimination of 103 unavailable values) across the Northern and Southern hemispheres was organised employing the Nestedness Temperature Calculator (Atmar and Patterson 1995). One hundred seven ubiquitous pathogen species were eliminated from the database because the information they contained was entirely redundant with that of the most ubiquitous species already present in the matrix. The matrix of species presence/absence provides distributional information about which species occurs in which countries.

#### GLIMs

We employed GLIMs (Crawley 1993; Venables and Ripley 1999) from the S-Plus statistical package (Venables and Ripley 1999) to identify and characterize the effects of potential independent parameters and their interaction terms on PID species richness, which is the total number of human diseases known within the boundary limits of each country. It has been argued that species richness increases with increasing area sampled (Hawkins and Porter 2001; but see Rohde 1997). Therefore, we included total surface area per country (in square kilometers) in our analyses, in order to control for its effect in the multivariate analysis. Similarly, we considered human population size and human population density per country (in persons per square kilometer), both highly colinear with surface area, as possible explanatory factors, since the number and density of human hosts may also influence parasite species richness (Anderson and May 1991; Guégan et al. 2001). In addition, we considered a variety of environmental, demographic, and economical factors. Variables selected as environmental factors for each country were (1) continent, (2) hemisphere, (3) whether the country was insular or continental, (4) percentages of arable land, permanent pastures, permanent crops, irrigated lands, forest woodlands, and “other,” (5) mean latitude coordinate, centered at the country barycenter (in minute degrees), and (6) mean longitude (in minute degrees) from the Greenwich Meridian.

Variables selected as demographic factors were (1) human population size, (2) human population density (persons per square kilometer), (3) human birth rate (births/1,000 people/year), (4) human death rate (deaths/1,000 people/year), and (5) annual population growth rate (average annual percent change in the population, resulting from a surplus or deficit of births over deaths and the balance of migrants entering and leaving a country).

We employed the gross national product (per capita in United States dollars) as the economic factor, which is the value of all final goods and services produced within a nation in a given year, plus income earned by its citizens abroad, minus income earned by foreigners from domestic production. We also selected a few other variables linked to particular landscape practices (percentages of arable land, permanent pastures, permanent crops, irrigated lands, forest woodlands, and “other”), which were supposed to interact with the production of the nation. Data were collected from The World Factbook 2001 on the Internet (http://www.cia.gov/cia/publications/factbook) and from the appendix of Scott and Duncan (1998).

To relate richness to environmental factors, we employed a GLIM with a Poisson error and a log link function (see Wilson and Grenfell 1997). Factors and their interaction terms were selected by a backward stepwise elimination procedure from the general model according to the Akaike criterion (Crawley 1993; Burnham and Anderson 2002). Deviances were compared using χ^2^ statistics.

#### Spatial autocorrelation analysis

When data suggested nonlinear trends, explanatory variables were transformed and fitted again to improve their contribution to the models. Since close geographical neighbors (i.e., two countries sharing a boundary) probably also share common PID species, simple cross-country comparisons could include spatial autocorrelation artefacts (Manly 1991). To test whether this influenced our regressions, we employed Monte Carlo simulations to calculate Moran's index *(I)* between the matrix of PID species richness and the matrix of distances across the 224 countries (Manly 1991; Guégan and Hugueny 1994). The *I* value is bound between −1 and +1, with 0 indicating no spatial autocorrelation, and +1/−1 indicating a strong positive/negative autocorrelation, respectively. We first computed the correlation coefficient based on all pairs of neighboring countries, and we randomly estimated 99 coefficients each time, permuting the matching countries. The decision rule, ensuring significance at α = 0.01, consisted in rejecting the null hypothesis of the absence of spatial correlation if the correlation coefficient obtained for nonpermuted data was maximum among all 100 coefficients. The calculation of *I* using Monte Carlo simulations indicated no strong spatial autocorrelation (*I*_0_ = 0.08 equals *I*_s_ = 0.11 at α = 0.01), suggesting that the close similarities between PID species richness and composition observed between neighboring countries conforms to the latitudinal diversity gradient.

#### Nestedness analysis

We also employed Monte Carlo simulations (Manly 1991; Guégan and Hugueny 1994) to evaluate PID spatial organization at the largest scale. We used the data matrix of presence/absence values for 229 different pathogen species of the total dataset comprising 224 countries. We assessed the degree of nestedness of the system using two different, but complementary, analysis programs: (1) Nestedness (Guégan and Hugueny 1994) and (2) Nestedness Temperature Calculator (Atmar and Patterson 1995). Nested diversity patterns are identified when species found in depauperate communities represent nonrandom subsets of progressively richer communities (Gaston and Blackburn 2000; Poulin and Guégan 2000). In procedure 1, pathogen species were either selected with uniform probability (null model R_0_) or with a probability proportional to their incidence (R_1_) (Guégan and Hugueny 1994), whereas in procedure 2 we tested the null model R_00_ (Poulin and Guégan 2000; see also Cook and Quinn 1998; Wright et al. 1998; Gaston and Blackburn 2000). Nestedness Temperature Calculator generates simulated null matrices without either row or column constraints (hence “00”); only the total number of presences is fixed at the observed value. All three null hypotheses assume that sites are independent of one another (Wright et al. 1998).

According to the procedure adopted by the Nestedness Temperature Calculator (see Atmar and Patterson 1995), the matrix is first “packed” into a state of maximum nestedness, reordering rows and columns. By convention, the most species-rich country is placed along the top row, and the most widely distributed species is placed in the leftmost column, so as to concentrate presences in a corner of the matrix, and to minimize unexpected species absences and presences as in theoretical Figure 1A. This will make differences in PID species distribution across countries readily perceivable. Moreover, not all unexpected species presences and absences are of equal informational value, and this must be taken into account. As we move away from the corner, where cells are most likely to be occupied, unexpected absences and presences begin to appear. The occurrence boundary lines (black exponential curves in Figure 2B) are based on the distribution of unexpected species' presences and absences within the matrix. These curves determine the hypothetical boundary between the occupied area of the matrix and the unoccupied area. A color scale indicates the probability of a cell's occupancy. Nestedness Temperature Calculator also includes a Monte Carlo component to assess the statistical assurance that the parent data matrix was not randomly generated. To assess that probability, 1,000 randomized permutations were drawn to determine a baseline expectation. The result is a histogram representing the 1,000 “temperature” values obtained after permutations (Figure 2C). A black arrow indicates the “temperature” value observed with our master matrix. Lastly, the probability of obtaining this value by random is calculated.

## Abbreviations

GLIM: generalized linear model
PID: parasitic and infectious disease

## References

[pbio-0020141-Allen1] Allen AP, Brown JH, Gillooly JF (2002). Global biodiversity, biochemical kinetics, and the energetic-equivalence rule. Science.

[pbio-0020141-Anderson1] Anderson RM, May RM (1991). Infectious diseases of humans: Dynamics and control.

[pbio-0020141-Ashford1] Ashford RW (2000). Parasites as indicators of human biology and evolution. J Med Microbiol.

[pbio-0020141-Ashford2] Ashford RW, Crewe W (1998). The parasites of *Homo sapiens* An annotated checklist of the protozoa helminths and arthropods for which we are home.

[pbio-0020141-Atmar1] Atmar W, Patterson BD (1995). The nestedness temperature calculator: A visual basic program, including 294 presence-absence matrices. Chicago: AICS Research. Available: http://aics-research.com/nestedness/tempcalc.html via the Internet. http://aics-research.com/nestedness/tempcalc.html.

[pbio-0020141-Brown1] Brown JH (1995). Macroecology.

[pbio-0020141-Burnham1] Burnham KP, Anderson DR (2002). Model selection and multimodel inference: A practical information-theoretic approach.

[pbio-0020141-Chown1] Chown SL, Gaston KJ (2000). Areas, cradles and museums: The latitudinal gradient in species richness. Trends Ecol Evol.

[pbio-0020141-Combes1] Combes C (1995). Interactions durables: Écologie et évolution du parasitisme.

[pbio-0020141-Cook1] Cook RR, Quinn JF (1998). An evaluation of randomization models for nested species subsets analysis. Oecologia.

[pbio-0020141-Crawley1] Crawley MJ (1993). GLIM for ecologists.

[pbio-0020141-Curtis1] Curtis TP, Sloan WT, Scannell JW (2002). Estimating prokaryotic diversity and its limits. Proc Natl Acad Sci U S A.

[pbio-0020141-Gaston1] Gaston KJ, Blackburn TM (2000). Pattern and process in macroecology.

[pbio-0020141-Guegan1] Guégan JF, Hugueny B (1994). A nested parasite species subset pattern in tropical fish: Host as major determinant of parasite infracommunity structure. Oecologia.

[pbio-0020141-Guegan2] Guégan JF, Thomas F, Hochberg ME, de Meeûs T, Renaud F (2001). Disease diversity and human fertility. Evolution.

[pbio-0020141-Hanski1] Hanski I (1982). Dynamics of regional distribution: The core and satellite species hypothesis. Oikos.

[pbio-0020141-Hawkins1] Hawkins BA, Porter ER (2001). Area and the latitudinal diversity gradient for terrestrial birds. Ecol Lett.

[pbio-0020141-Hawkins2] Hawkins BA, Field R, Cornell HV, Currie DJ, Guegan J-F (2003). Energy, water, and broad-scale geographic patterns of species richness. Ecology.

[pbio-0020141-Hill1] Hill JK, Thomas CD, Huntley B (1999). Climate and habitat availability determine 20th century changes in a butterfly's range margin. Proc R Soc Ser B-Bio.

[pbio-0020141-Hillebrand1] Hillebrand H, Watermann F, Karez R, Berninger UG (2001). Differences in species richness patterns between unicellular and multicellular organisms. Oecologia.

[pbio-0020141-Huston1] Huston MA (1999). Local processes and regional patterns: Appropriate scales for understanding variation in the diversity of plants and animals. Oikos.

[pbio-0020141-Kaufman1] Kaufman DM (1995). Diversity of New World mammals: Universality of the latitudinal gradients of species and bauplans. J Mammal.

[pbio-0020141-Lindgren1] Lindgren E, Gustafson R (2001). Tick-borne encephalitis in Sweden and climate change. Lancet.

[pbio-0020141-Lomolino1] Lomolino MV (1996). Investigating causality of nestedness of animal communities: Selective immigrations or extinctions?. J Biogeogr.

[pbio-0020141-Manly1] Manly BFJ (1991). Randomization, bootstrap and Monte Carlo methods in biology.

[pbio-0020141-Nee1] Nee S (2003). Unveiling prokaryotic diversity. Trends Ecol Evol.

[pbio-0020141-Patterson1] Patterson BD, Atmar W (1986). Nested subsets and the structure of insular mammalian faunas and archipelagos. Biol J Linn Soc.

[pbio-0020141-Patterson2] Patterson BD, Brown JH (1991). Regionally nested patterns of species composition in granivorous rodent assemblages. J Biogeogr.

[pbio-0020141-Pianka1] Pianka ER (1966). Latitudinal gradients in species diversity: A review of concepts. Am Nat.

[pbio-0020141-Poulin1] Poulin R (1998). Evolutionary ecology of parasites: From individuals to communities.

[pbio-0020141-Poulin2] Poulin R, Guégan JF (2000). Nestedness, anti-nestedness, and the relationship between prevalence and intensity in ectoparasite assemblages of marine fish: A spatial model of species coexistence. Int J Parasitol.

[pbio-0020141-Rahbek1] Rahbek C, Graves GR (2001). Multiscale assessment of patterns of avian species richness. Proc Natl Acad Sci U S A.

[pbio-0020141-Rohde1] Rohde K (1992). Latitudinal gradients in species diversity: The search for the primary cause. Oikos.

[pbio-0020141-Rohde2] Rohde K (1997). The larger area of the tropics does not explain latitudinal gradients in species diversity. Oikos.

[pbio-0020141-Rosenzweig1] Rosenzweig ML (1995). Species diversity in space and time.

[pbio-0020141-Roy1] Roy K, Jablonski D, Valentine JW, Rosenberg G (1998). Marine latitudinal diversity gradients: Tests of causal hypotheses. Proc Natl Acad Sci U S A.

[pbio-0020141-Scott1] Scott S, Duncan CJ (1998). Human demography and disease.

[pbio-0020141-Shaw1] Shaw MR, Hochberg ME (2001). The neglect of parasitic Hymenoptera in insect conservation strategies: The British fauna as a prime example. J Insect Conserv.

[pbio-0020141-Stevens1] Stevens GC (1989). The latitudinal gradient in geographic range: How so many species coexist in the tropics. Am Nat.

[pbio-0020141-Stevens2] Stevens GC (1992). The elevation gradients in altitudinal range: An extension of Rapoport's latitudinal rule to altitude. Am Nat.

[pbio-0020141-Stevens3] Stevens RD, Cox SB, Strauss RE, Willig MR (2003). Patterns of functional diversity across an extensive environmental gradient: Vertebrate consumers, hidden treatments and latitudinal trends. Ecol Lett.

[pbio-0020141-Venables1] Venables WN, Ripley BD (1999). Modern applied statistics with S-PLUS, 3rd ed.

[pbio-0020141-Wilson1] Wilson K, Grenfell BT (1997). Generalized linear modelling for parasitologists. Parasitol Today.

[pbio-0020141-Wright1] Wright DH, Patterson BD, Mikkelson GM, Cutler A, Atmar W (1998). A comparative analysis of nested subset patterns of species composition. Oecologia.

